# 

**Published:** 1881-02

**Authors:** 


HERMAN MYNTER, M. D.

### CONTENTS:

PAGE.

ORIGINAL COMMUNICATIONS:

Stricture of the Trachea. By Herman Mynter, M. D., . . . . . . . . 289

CLINICAL REPORTS:

Prof. E. M. Moore's Surgical Clinic at the Buffalo General Hospital. Reported by F. Peterson, M. D., . . . . . . 296

A Case of Angular Curvature of Spine, preceded by Lumbar Abscess, treated by Leather Brace, etc. Reported by Bernard Bartow, M. D., . . . . . . . 299

Cancer of Liver and Pancreas, Large Collection of Calculi in Gall-Bladder. Reported by Thos. Lothrop, M. D., . . 303

Compound Comminuted Fracture of the Frontal Bone — Trephining — Recovery. Reported by James S. Smith, M. D., . 306

TRANSLATIONS:

A Confinement with Rupture of the Uterus. From the German by P. W. Van Peyma, M. D., . . . . . . . . . 307

SELECTIONS:

A Clinical Lecture on the Value of Partial Intoxication in the Prevention of Shock during Operations. By Stephen Smith, A. M., M. D., . . . . . . . 311

Artificial Nourishment of Sucklings in the First Ten Weeks of Life, . . . . 314

Gastric Ulcer and Cancer of the Stomach, . 316

The Chloroform Death-Bill for Twelve Months. By Ernest H. Jacob, M. D., . 317

Antiseptic Midwifery, . . . . . . 319

Tendon-Reflex, . . . . . . . 320

A Spinal Root of the Optic Nerve, . . 320

Reflex Cough, . . . . . . . 321

Quinine with Nervous Sedatives, . . 321

Cannabis Indica in Hemicrania, . . . 322

The Specific Agent of Typhoid Fever, . . 322

Eczema at the Umbilicus, . . . . . 323

Hyoscyamia, . . . . . . . . 323

Wickersheimer's Preparation, . . . . 324

EDITORIAL:

The Forthcoming Harvest of Doctors—Some Suggestions, . . . . . . . 325

Alumni Association, Buffalo Medical College, . . . . . . . . . 327

The Medical Law—Its Enforcement, . . 328

REVIEWS, . . . . . . . 329

